# A Qualitative Exploration of Multi-level Factors that Support Effective Community Health Worker-Social Worker Collaboration

**DOI:** 10.21203/rs.3.rs-6733746/v1

**Published:** 2025-06-13

**Authors:** Nidhi Monga Nakra, Liana J. Petruzzi, Alexander B. Diller, Julie Smithwick, Lily Lee, Geoff Wilkinson, Joshua Collier, Sarah Chang

**Affiliations:** Johns Hopkins University; American University; American University; University of South Carolina; Knowledge Transfer & Exchange Strategies, LLC; Boston University; National Center for Farmworker Health; Boston University

**Keywords:** Community Health Workers, Social Workers, patient care team, interdisciplinary communication, health equity

## Abstract

**Background:**

Interdisciplinary collaboration is critical for improving healthcare delivery through coordinated care and streamlined healthcare navigation. Community health workers (CHWs) and social workers (SWs) are uniquely positioned to address the needs of individuals with complex social and health challenges. Despite the integration of CHWs and SWs into health and community settings, there is a paucity of literature on what facilitates successful collaboration between these two workforces. This qualitative study, conducted from April 2022 to June 2023, explores multilevel factors related to CHW-SW collaboration in health and community settings.

**Methods:**

We conducted eight, 90-minute virtual focus groups with CHWs (n = 20) and SWs (n = 17) collaborating in four healthcare and community health settings across the United States (California, Texas, New Jersey, and South Carolina). Focus groups were conducted between April 2022 and June 2023.

**Results:**

Themes were thematically organized according to the socio-ecological model. Individual and relationship-level factors included: roles and scopes of practice, communication, mutual respect, supportive supervision, and power dynamics. Organizational and community-level factors comprised: commitment to equity, leadership buy-in, standardized training, clear workflows, and shared documentation and physical space. Societal-level factors included: power dynamics, supportive policies and sustainable funding.

**Conclusions:**

Findings highlighted that CHW-SW collaboration can promote patient-centered care and address social determinants of health when both workforces are well integrated in healthcare systems. Key organizational commitments, community rapport, and relational dynamics should be established to optimize interdisciplinary collaboration and advance health equity.

## Background

Community health workers (CHWs) and social workers (SWs) play pivotal, overlapping, and distinct roles in addressing individual and community needs (1). CHWs often serve as the first point of contact within health systems, leveraging their lived experiences, cultural understanding, community knowledge, and rapport to build trust, promote health education, and connect individuals with essential services (2). Meanwhile, SWs contribute clinical expertise in psychosocial assessment, emotional support, and behavioral health management (3,4). Despite increasing integration of both professions into health and community settings, little is known about how organizational and relational dynamics shape collaboration between CHWs and SWs, or what conditions enable these teams to function effectively. Further research in these areas could improve interdisciplinary integration as well as healthcare delivery more broadly.

The COVID-19 pandemic and ongoing structural health inequities underscored the urgency of optimizing all available human resources to address community needs, especially amid healthcare and nursing staffing shortages (5). Disruptions in traditional practices prompted healthcare workers to rapidly adjust roles, adopt flexible service models, and develop new ways of coordinating care. Simultaneously, systemic issues such as uneven resource allocation, ingrained professional hierarchies, and limited funding streams persisted, straining interprofessional relations and collaboration(6,7). These dynamics are particularly evident in settings where both CHWs and SWs must navigate changing health policies, increased client needs, and complex social and economic contexts. Therefore, this study explores the multilevel factors that influence CHW-SW collaboration in health and community settings, with the goal of informing more integrated, equitable approaches to care delivery.

## Methods

### Study Design

We used an exploratory qualitative design to identify elements of effective collaboration and integration among CHWs and SWs at health and community organizations. This study was reviewed by the University of Texas at Austin’s Institutional Review Board (IRB) and deemed exempt. However, to ensure that all participants were informed about the study procedures and risks, all participants reviewed and signed electronic consent forms prior to completing the survey and/or focus group.

### Sampling

First, we identified eligible organizations based on recommendations from the Coalition for CHW-SW Collaboration and snowball sampling. Then, we contacted organizations to confirm CHW-SW collaboration and integration, and to assess their interest, capacity and staff size. We utilized stratified sampling to include health and community organizations across diverse regions and populations.

Four organizations were recruited from New Jersey, Texas, South Carolina and California that work with various populations (social and medically complex individuals, cancer patients and survivors, perinatal individuals and families, and Medicaid health plan members). Organizations were eligible if they had integrated both CHWs and SWs into their services and had at least 4 CHWs and 4 SWs for adequate focus group sampling.

### Study Procedures

We used purposive, non-probabilistic sampling to recruit CHWs and SWs. Our team emailed a flyer to 24 organizations, inviting them to participate in a study about CHW-SW collaboration and integration. Six organizations responded, but two were excluded due to insufficient sample size for a final sample of four organizations. For more details, refer to Table 1.

CHWs and SWs provided consent prior to a brief online survey via REDCap (8,9). All survey respondents were invited to join a focus group discussion. Of the 39 survey participants, 34 participated in focus groups, which were disaggregated by organization and role (SW or CHW) to minimize potential power dynamics. Groups consisted of three to six participants.

A 12-question, semi structured interview guide focused on the following topics: organizational scope, population served, and services provided; CHW-SW collaboration and integration; and the influence of COVID-19. Questions included “How do CHWs and SWs collaborate at your organization?” and “What factors facilitate SW-CHW collaboration?” A script was used at the beginning of each focus group to clarify the study’s purpose, confidentiality agreement and provide an opportunity to ask questions.

Between April 2022 and June 2023, the second author facilitated eight, 90-minute focus groups virtually. The focus group participants received a $100 electronic gift card. Focus groups were conducted in English, audio-recorded via Zoom, and transcribed verbatim. The first and second authors checked the transcripts for accuracy and proper identification of participants. The data were then deidentified and uploaded into NVivo (10).

### Analysis

Transcripts were coded and analyzed deductively and inductively. Our deductive approach was guided by our conceptual framework for effective CHW-SW collaboration and integration (11). We incorporated key concepts such as standardized training, defined roles and shared physical location into the codebook. We also employed inductive thematic analysis to identify new concepts, recurrent patterns, and nuanced subthemes (12,13).

Two primary coders (first and second authors) independently reviewed two focus groups (one with CHWs and one with SWs) and coded them line-by-line to identify preliminary themes. After each focus group was independently coded, the coders met and reviewed the transcripts line-by-line to reach consensus. These preliminary codes informed a second round of independent coding of two additional focus groups (one with CHWs and one with SWs) to ensure adequate inter-rater reliability. The first author then coded the remaining four focus groups; however, the primary coders still met after each transcript was coded to discuss ambiguous quotes and consider any codebook changes.

Codebook development was an iterative process of coding a transcript, consultation and consensus, updating the codebook, and reviewing previous transcripts to ensure new codes were added. Saturation was measured by calculating the percentage of new codes added to the codebook after each transcript (14). We used a base number of 5 transcripts, a run length of 2 transcripts and a new information threshold of 5%. The codebook stabilized after five transcripts, indicating that conceptual saturation had been reached.

### Member Checking

We conducted member checking with CHWs, SWs, and public health professionals throughout data collection and analysis. As transcripts were coded, initial themes were shared with the Coalition for CHW-SW Collaboration, a group of 60 members from 11 states, representing health and community organizations as well as academic institutions (11). The Coalition met quarterly to review the study findings, offer insights that enhanced the richness of the data, and clarify any inconsistencies. Members who were unable to participate in the meetings were invited to offer feedback via an online survey.

## Results

### Demographics

Table 2 summarizes the demographics for participants who completed the qualitative focus groups. The participants were predominantly female in both groups. All CHWs identified as Black (52%) or Hispanic/Latinx (48%), whereas SWs were more racially and ethnically diverse (Black: 37%, White: 25%, Hispanic/Latinx: 19%, and Asian: 19%). In terms of language, 57% of CHWs spoke English only, compared to 75% of SWs. Regarding education, 87% of SWs held graduate degrees compared to 13% of CHWs.

### Qualitative Findings

**Figure 1:**
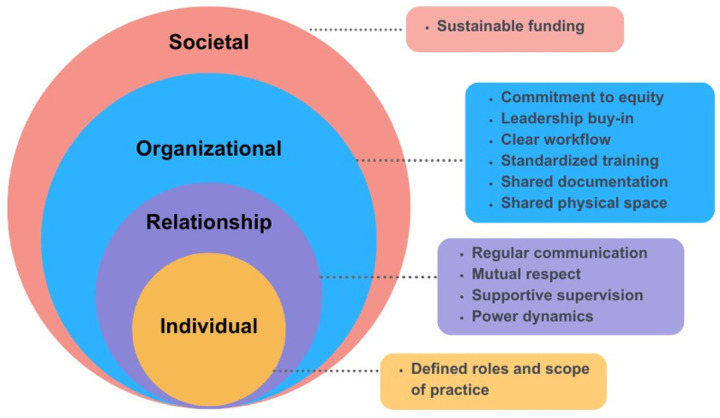
Factors that support CHW/SW collaboration across system levels

This study identified multilevel factors that influence CHW-SW collaboration, and organized themes according to the socioecological framework at individual, relationship, organizational, and societal levels (15–17). Of note, we utilize the term “clients” within the body of the paper; however, quotes are reported verbatim. Figure 1 and Table 3 provide an overview of all themes.

### Individual-Level Factors

We defined individual-level factors as characteristics of personal and professional identity that influence effective collaboration. *Defined roles and scopes of practice* consistently emerged as critical components of CHW-SW collaboration. When roles were clearly delineated, CHWs and SWs complemented one another; they worked efficiently and synergistically to provide holistic care. While roles are often shaped by organization or context–such as setting or population served–scopes of practice are defined by national associations and licensing or certifying bodies (18,19).

Across organizations, CHWs and SWs were committed to advancing patient-centered care and health equity. Both CHWs and SWs engaged in care coordination, resource referrals, capacity building, and advocacy, yet their roles differed based upon professional scope and acuity of client needs. For example, CHWs primarily focused on community outreach and engagement, systems navigation, and trust-building. They were described as “foot soldiers,” “a voice for those who don’t have one,” a “one-stop-shop,” and the “primary point person.” As local experts, they were engaged when clients presented with socially complex concerns, including housing instability, chronic illness or disability. Alternatively, SWs leveraged their clinical expertise to conduct biopsychosocial assessments, make diagnoses, and provide behavioral support. SWs routinely managed high-risk cases involving mental health, substance use, medical conditions or safety concerns.

When CHW and SW roles were clearly defined within program design and referral workflows, teams were aligned to address complex client needs and collaborate effectively:

“That is where we will delegate more of the social determinants of health…if there is a food insecurity or housing insecurity, we will document that for the CHW to follow up on…So the roles are delegated and clearly defined”

(SW, Org 1).

Without this clarity, CHWs and SWs experienced confusion about their responsibilities, impeding them from working efficiently. This arose from a lack of understanding internally from organizational leadership, management, or team members.

At times, role confusion extended beyond the organization, especially for CHWs navigating relationships with community partners: “I think there is that sense that inside [this org] I feel good and supported, but when I sometimes have to go outside these spaces, I’m suddenly like, people might not even know what the community health worker is” (SW, Org 4). Therefore, role clarity within organizations and with community partners are both crucial for CHW-SW collaboration.

Additionally, mistrust of health and social service systems presented challenges for SWs, particularly within communities of color where SWs have historically been associated with child welfare: “I don’t want someone coming in, you know? They may take my child, or whatever. So, calming that fear helps when you don’t use the word ‘social worker’” (CHW, Org 3). In these cases, CHWs helped bridge the trust gap by leveraging their community identity and building rapport with clients before connecting clients with SW services.

### Relationship-Level Factors

Relationship-level factors reflected the interpersonal components that influence CHW-SW collaboration, including regular communication, mutual respect, supportive supervision, and power dynamics.

*Regular communication* facilitated CHW-SW collaboration through team “huddles,” multidisciplinary meetings, and case consultations, bringing diverse partners together to review and address client needs holistically:

“We also meet every week to discuss our patient panel. We have our medical director there, we have the social worker there, everyone’s involved, everyone’s hands on, everyone’s hearing the story, and everyone shares their input”

(CHW, Org 4).

These intentional spaces encouraged inclusive dialogue, empowering CHWs and SWs to share openly and feel recognized: “I think being able to communicate in an open, respectful way where you feel valued and appreciated, as a worker…as a person. I feel like that’s something that stands out from this team…that I value so much” (SW, Org 1).

Regular communication was especially critical for teams that worked in different, often siloed settings. Joint home visits presented clients with a unified approach to care coordination and encrypted EMR chat tools facilitated seamless follow-through between team members:

“We communicate like a private chat with the CHWs, every day. They send the cases they considered, [did] the assessment, need a follow up, more help because the social determinants of health were issues…After that, we can chat directly with the SW to ask more questions or receive some feedback”

(CHW, Org 1).

While routine, structured communication promoted teamwork, communication challenges occasionally emerged, straining relational dynamics and disrupting client care. One CHW reflected on a conversation with an SW who resisted organizational practices that differed in scope from her previous experiences; this interaction created some tension internally:

“There’s a lot of experience that they bring…but not every place is the same…there’s certain things that we do here - process-wise, system-wise, notation-wise. Sometimes… they fight back, and they say ‘oh, well here, we did it like this’”

(CHW, Org 1).

Unexpected disruptions further impacted communication, leading to missed opportunities for case consultation, gaps in coordination, and some client concerns going unaddressed. This was particularly challenging during the COVID-19 pandemic when team members were absent due to illness and in-person activities were interrupted: “It wasn’t like we could sit around the table that we normally do and discuss the case” (SW, Org 3).

Inefficient communication with external partners was also a challenge. For example, while CHWs and SWs frequently received outside referrals, clients were not always well informed: “A client who was referred to us from [hospital system] came to me, and when I contacted them, they said, ‘Who are you? I don’t know what you want. What are you trying to sell me?’” (SW, Org 2). These challenges not only impacted the client experience but also limited the ability of CHWs and SWs to build trust and function as a care team.

*Mutual respect* between CHWs and SWs was another important factor. Recognizing and appreciating each other’s professional value and expertise encouraged trust, cohesion, and shared purpose. SWs regarded CHWs as “field experts” and “connections to our community,” commending their ability to build community rapport, offer historical knowledge, and navigate complex systems: “Two of our CHWs are cancer survivors…they understand what it means to be on the client side and…on the provider side. That makes us well positioned to be an effective group” (SW, Org 2). Similarly, SWs were valued for their diverse experiences, advanced education, and clinical expertise, which provided CHWs with alternate perspectives when managing complex client needs:

“I work very closely with the social workers because they are a wealth of resources. They know all kinds of things, and usually what they don’t know, they can find. For the higher need clients…I do, for sure, seek out their guidance”

(CHW, Org 2).

Encompassing mutual respect was a demonstrated willingness to learn. CHWs and SWs acknowledged the limits of their respective training and how they could more effectively collaborate with the guidance of others:

“I just feel like it’s good to have the support of another team member when working with a client, because, like they say, we don’t know everything. We’re not experts in every area. So, just having people who have different experiences is a good thing”

(SW, Org 3).

Furthermore, there was a general perception that an “all hands on deck” approach promoted successful client outcomes.

In terms of *supportive supervision*, participants emphasized two factors: who was delivering the supervision and how it was being delivered. It was not uncommon for CHWs or SWs to be managed by nurses or physicians with limited training or understanding of CHW/SW roles and scopes of practice. This mismatch can recreate hierarchical dynamics, where CHWs and SWs feel undervalued. Participants highlighted the importance of role concordance between CHWs and SWs and their respective supervisors to support this awareness.

In cases where SWs supervised CHWs, several practiced “reflective supervision,” where they openly acknowledged professional “privilege” and reflected on social inequities shaped by race, ethnicity, or immigration status. They described how CHWs frequently faced systemic barriers to education and employment, and were sometimes overlooked in workplaces, particularly health settings. SWs leveraged their status within these systems to counter power imbalances, promote the community health worker discipline, and empower CHWs to grow personally and professionally: “A lot of the work that I’ve done in supervision is …helping develop confidence in that space and that…you don’t have to change who you are to be in that space. What we want to do is change the space so that your voice is heard there and respected there” (SW, Org 4).

Furthermore, access to career advancement opportunities improved work morale and promoted the retention of care team members. In one setting, a supervising SW recognized the systemic barriers to compensation for CHWs and advocated for their promotion:

“I think we do people a disservice when it’s like…I want to grow, or I want more pay or I want a different title. If you’re not giving them the training or the skills, it’s not effective at all. I don’t want to see that happen to my staff”

(SW, Org 4).

Finally, *power dynamics* emerged in ways that were both collaborative and limiting–reflecting efforts to share power while acknowledging persistent hierarchies. For example, SWs used their credibility to advocate for CHWs in interdisciplinary settings. One SW described consciously stepping back in community interactions to ensure the CHW–“who was actually doing the work”–could directly engage with partners. Others spoke about amplifying CHW voices within coalitions, healthcare systems, and policy settings to bring visibility to their value as trusted community members with lived experience. Nevertheless, power imbalances persisted both within CHW-SW teams and in client interactions. Despite their complementary roles, CHWs viewed themselves differently in relation to SWs, particularly in terms of training, pay, and licensure: “Just a feeling of the [CHW] role might not be as respected in other spaces, especially in medical spaces…where there can be these really entrenched hierarchies and power dynamics” (SW, Org 4). These dynamics also extended to healthcare settings, wheretraditional norms of authority influenced how CHWs were perceived.

### Organizational-Level Factors

Organizational-level factors included the structures, policies or practices that shaped CHW-SW collaboration. Factors included commitment to equity, leadership buy-in, clear workflows, standardized organizational training, and shared documentation and physical spaces.

*Commitment to equity* was defined by equity-oriented practices that addressed upstream social determinants and fostered inclusive workplaces. This collaborative culture was described as follows:

“We’re all collaborating together in a way that is very different. I’ve seen other places where tasks are stratified among sort of hierarchies or roles. And at [Org 4] it is just so collaborative, and everyone can sort of see the big picture…I think because of that, everyone feels more buy-in to the work as well”

(SW, Org 4).

*Leadership buy-inwas* essential to creating supportive environments that empowered CHWs and SWs to collaborate. Participants appreciated how effective leaders prioritized staff professional development through skill-building opportunities, and intentionally focused on staff well-being: “They want to know more about [us] personally. And I love that about it. We’re able to express how we feel…what more training we want or what other things we want to tap into” (CHW, Org 4). Leaders also engaged CHWs in decision-making that shaped organizational programs and policies, establishing a sense of shared ownership: “This new program is launched because we heard from you, the CHWs, about a need. So, it continues to inform the work” (SW, Org 4). Finally, leaders encouraged autonomous work, which empowered teams to invest the time needed to support their clients comprehensively.

*Clear workflows* and defined referral processes facilitated coordination between CHWs and SWs and strengthened rapport with community partners. When CHWs and SWs were effectively integrated within organizations, tasks were efficiently delegated; they understood when to hand off to a CHW or SW or refer out for additional support. One SW described the process as collaborative: “Depending on what the needs of the patient is, that’s how we decide who could be the primary and we let the patients know…we’re part of a team. And each team member plays a different role” (SW, Org 1).

When workflows were unidentified, participants expressed confusion about how to manage cases and over which tasks fell under whose responsibilities, resulting in duplicative efforts or missed opportunities to provide care:

“Just handed [the case] off, and then again the repeating, and then they’re not getting the care that they need or getting their problem solved because they’re being passed around from everybody, because that person that they get to doesn’t know what they’re doing”

(CHW at Org 1).

Confusion was common in settings where CHWs were newly integrated, and roles were loosely defined.

Another key factor was *standardized organizational training*, which ensured that teams were aligned on processes and practices. One site explicitly included all staff in training, regardless of role or seniority: “The professional development through [Org 4] is just not stratified…It is like the CEO and leadership sitting side by side in training with all levels of the organization” (SW, Org 4). In addition, some organizations prioritized cross-training, with CHWs and SWs collaboratively sharing insights from external training sessions to promote best practices and patient-centered care:

“If the CHWs went to a training, and they gained a lot of knowledge, they would come back to the office and share around the table…if we went to a training, we would come back and share with them what we learned. Keeping that collaboration…keeping each other educated”

(SW, Org 3).

In contrast, a lack of standardized traininghindered team cohesion and left some participants feeling unprepared in their roles. One CHW, for instance, noted the absence of a formal onboarding process:

“When I started it was just like, ‘here you go, do some intakes.’ I was like ‘oh my gosh I’ve never done this before.’ So, we are working towards…getting that a little more standardized”

(SW, Org 2).

*Shared documentation systems* were essential tools through which CHWs and SWs could effectively coordinate care. Internal tracking systems equipped teams with real-time access to client information and ensured that assessments, diagnoses, care plans, and referrals were documented and monitored. CHWs and SWs routinely used these resources to communicate about clients, follow-up on tasks, and hand-off care when needed:

“We have a tracking system where we can assign a task to a social worker or another community health worker. This way it pops up. So, there’s a few ways that we can assign tasks and communicate and push these out to the necessary providers”

(CHW, Org 2).

In one organization, Health Information Exchanges enhanced interoperability, allowing teams to communicate with outside hospitals and providers, access medical records, and flag priority clients in need of critical services or resources. Alternatively, when systems were not well integrated, participants described navigating multiple platforms as “time-consuming” and a “hindrance.”

Finally, CHWs and SWs valued *shared physical space*. Being situated in close proximity to one other, facilitated routine communication, continuity of care, and team cohesion: “We’re blessed that [we are] in the same office with our social worker…where we can communicate, pick up the telephone and call them because we are all staffed together…And that helps make that coordination of care easier” (CHW, Org 3). These factors highlight the need for organizational leadership to establish inclusive structures that encourage shared decision-making and harmonize the CHW-SW relationship.

### Societal-Level Factors

Societal-level factors encompassed social, cultural, political and historical components that influenced how CHWs and SWs performed their respective duties and responded to community needs. Access to *sustainable funding* for CHW-SW collaboration was a primary theme across focus groups. For example, Enhanced Care Management through state Medicaid supported meaningful CHW-SW collaboration. This benefit enabled teams to address all aspects of client health and well-being collaboratively, including key social determinants like income and housing. This model both expanded access to services and broadened one SW’s perspective on interdisciplinary teams:

“Prior to joining this care management team, I just did straight behavioral health…I had never really worked as part of a team before, like interdisciplinary team…I really like the concept and I have seen it work…I have seen patients improve and with this collaboration”

(SW, Org 1).

Local foundations also funded CHW-SW collaboration. One participant described a grant that supports cross-sector collaboration to address clients’ multifaceted social, economic, and health challenges: “It’s not just a cancer issue—it’s a food insecurity issue, it’s a health literacy issue” (SW, Org 2).

## Discussion

As the need to address complex social determinants of health grows, interdisciplinary teams that improve service capacity and healthcare delivery have become increasingly important (20). This study explored key factors of effective CHW-SW collaboration—a promising, community-responsive approach to tackle health inequities. Findings illustrated that this collaboration is shaped by multi-level, intersecting factors, consistent with previous literature (21,22).

CHWs and SWs hold distinct yet overlapping roles on collaborative teams. While CHWs serve as trusted community connectors and system navigators, SWs provide clinical expertise and behavioral health support. Our study affirms that clearly defined roles and scopes of practice enhance CHW-SW collaboration by efficiently allocating tasks and minimizing duplicative efforts. These findings support prior research that emphasizes role clarity as a facilitator of successful cross-sector collaboration and service delivery (23,24). Although our data also reveal that role confusion persists both within organizations and with community partners, workforce development and standardized organizational training that includes CHWs, SWs, and leadership, can build awareness and recognition of both roles (25).

CHW-SW collaboration is reinforced by strong relational dynamics, including mutual respect, communication, and supportive supervision. When both workforces understand and value each other’s roles and skills, the contributions of each profession are elevated within the care team. Both CHWs and SW work together to provide diverse perspectives and expertise that uniquely address the clients’ needs. This shared decision-making—facilitated via case consultations and interdisciplinary care meetings—encourages transparent communication, cultivates team trust and streamlines care coordination to improve patient outcomes (26). Integrated documentation systems and access to each other’s notes further support shared decision-making, although navigating multiple systems remains a challenge. This is a known barrier for healthcare professionals who work across settings, highlighting the need for secure data interoperability not only to support CHW-SW collaboration, but to streamline communication and healthcare navigation (27,28). Finally, supportive supervision is an essential component of collaboration. Ideally, supervisors have a strong understanding of each role, including the continuing education expectations required to maintain certification–a condition supported by role concordance (29). Reflective supervision and power sharing further mitigate power imbalances and create equitable partnerships, findings that align with the Sunnybrook framework for interprofessional collaboration, which emphasizes shared competence, trust, and continuous reflection as cornerstones of effective teamwork (30).

Organizational culture, supported by inclusive leadership, lays a foundation for CHW-SW collaboration and is a critical component in reducing health disparities (31). Leaders that advocate for multiple perspectives, engage CHWs and SWs in decision-making, and invest in flexible care models promote a culture of collaboration. They can shift the organizational mindset toward systems-oriented, coordinated care that prioritizes integration and equity. Leadership buy-in, including leadership education and advocacy, is also critical for securing sustainable funding, standardizing training structures, and supporting professional development. When CHWs and SWs are supported by the organization or healthcare system and integrated into the care team, individuals who are most at-risk are able to access services (32). Finally, practical strategies like streamlining workflows and implementing shared communication tools and spaces can reinforce healthy interprofessional dynamics.

Systemic factors, including power balances between healthcare professions, continue to challenge interdisciplinary collaboration efforts. Our study points to a need for more sustainable funding and policy initiatives that promote equity at multiple levels—internally through pay parity and career advancement opportunities (25), and externally through comprehensive, equity-driven approaches to care (33). Such policies are essential to continuity of services and workforce stability, particularly for CHWs, whose pay has historically been low or even volunteer based. To mitigate power dynamics, these efforts can be supplemented by leadership support, mutual respect, open and transparent communication, support for greater autonomy and relational supervision (34,35).

Our study shows that implementing effective CHW-SW collaboration requires a multifaceted approach—leveraging diverse skill sets, fostering team-based behaviors, shifting organizational culture, and establishing structural support. By embedding these principles into practice, CHW-SW collaboration can be a powerful model for improving health services delivery and promoting positive outcomes through tailored resources and support. Furthermore, policies that advance care coordination, support funding for interdisciplinary teams, and promote pay parity prioritize equity; they transform how CHWs and SWs deliver care, while ensuring that they are justly compensated for their work.

## Limitations

This study has several limitations. First, organizations were identified through community referrals from the national Coalition on CHW-SW Collaboration including several authors. Therefore, the recruitment process may have introduced some bias in the data collected. However, the researchers who contacted the organizations, collected and analyzed the data were not previously familiar with the organizations. Second, while stratified sampling was used to recruit diverse organizations based on geographic location and health or community setting, the sample is not representative of all CHWs and SWs in these fields. Additionally, qualitative analyses are not generalizable to the entire population, highlighting the need for more robust quantitative research and national surveys to confirm and elucidate our findings. Finally, four participants did not complete the survey, and several declined to report their age, resulting in incomplete demographic data.

## Conclusions

Healthcare access is increasingly important within our current political climate. This research advances our collective understanding of what is necessary to build and sustain effective CHW-SW teams capable of ensuring access to quality healthcare, improving client outcomes and dismantling health inequities. By highlighting actionable strategies for organizations—such as specifying roles and scopes of practice, investing in cross-training, encouraging reflective supervision, and fostering supportive leadership—we offer guidance for enhancing interdisciplinary synergy. We also identify systemic challenges that persist at higher levels of the socioecological environment, pointing to the need for broader policy and structural changes that value and support the nuanced work of these professionals. CHW-SW collaboration is a promising model for addressing both immediate individual needs and the broader societal forces that shape health outcomes, thereby contributing to more just, accessible, and person-centered health services.

## Supplementary Files

This is a list of supplementary files associated with this preprint. Click to download.

- MultilevelFactorsCHWSWCollabTablesBMC.docx

## Figures and Tables

**Table 1:** Characteristics of Focus Group Organizations (N=37)

Organization	Type of Organization	Region of the United States	Population Served	Participant Distribution
Organization 1	Health (hospital, clinic, or health plan)	West – California	Medicaid health plan that serves individuals with chronic physical or medical health conditions	CHW: 6 SW: 3 Total: 9
Organization 2	Community	South – Texas	Low-income cancer patients, primarily Black and Latinx	CHW: 3 SW: 5 Total: 8
Organization 3	Community	East – South Carolina	Perinatal individuals and families in rural communities	CHW: 6 SW: 4 Total: 10
Organization 4	Community	North – New Jersey	Socially and medically complex individuals	CHW: 6 SW: 4 Total: 10

**Table 2:** Demographics of CHWs and SWs Completed Qualitative Focus Groups (N= 34)^1^

	CHWs (n= 18)	SWs (n= 16)
**Age (mean, SD)** ^2^	45.4 (10.4)	35.2 (9.9)
**Gender (% female)**	17 (94%)	10 (63%)
**Race/Ethnicity**		
Black	10 (52%)	6 (37%)
White, non-Hispanic	0 (0%)	4 (25%)
Hispanic/Latinx	8 (48%)	3 (19%)
Asian	0 (0%)	3 (19%)
**Language**		
English only	11 (61%)	13 (81%)
English & Spanish	7 (39%)	3 (19%)
**Education**		
High School/GED	5 (28%)	0 (0%)
Some College	5 (28%)	0 (0%)
College Degree	7 (39%)	2 (13%)
Graduate Degree	1 (5%)	14 (87%)
**Years as CHW or SW**		
Less than 2 years	5 (28%)	2 (13%)
2–5 years	7 (39%)	5 (31%)
6–9 years	2 (11%)	3 (19%)
10+ years	4 (22%)	6 (37%)

1 Qualitative sample demographics does not include 2 CHW participants and 1 SW participant who did not complete the survey

2 Based on n=28 as some participants declined to report

**Table 3:** Qualitative themes and subthemes from Community Health Workers and Social Workers (N= 37)

Theme	Illustrative Quote
***Individual Level Factors***	
**Roles and scopes of practice**	*CHW, Org 3*: “If they’re medically high risk, and they have any heart problems, previous losses, stillborn, we’re referring them to our social worker that specializes in maternal mortality. If they score high on the Edinburgh [Depression Screening]…if they’re previous with domestic violence, we’re connecting them to a social worker.” *SW, Org 1*: “There’s always been a history of some crossover between CHWs and SWs. In the past, there was a lot more confusion and we would have CHWs that would clearly identify themselves as social workers and we would have to say, ‘Well, you know, not technically but yes, you are helping with the resource piece of things.’
***Relationship Level Factors***	
**Communication**	*CHW, Org 4*: “If we’re not able to come to a conclusion for that person, we table it. We try to bring it back and try something else different…we’re learning and we navigate through it together… It makes you feel heard and makes you feel connected with the team. We may not always have the answers, but we [are] always going to come together.” *CHW, Org2*:“I had an intake in June… and then right after that I caught COVID. I’m just coming back to work now…I get an actual call from the client, and he said, ‘Nobody has called me.’ I said ‘Oh my gosh. Since June?’ I apologized to him, and I called the SW. It just fell through the cracks, nobody knows what happened.”
**Mutual Respect**	*CHW, Org 3*: “And sometimes we may not know something, and they may know something or vice versa. So we kind of collaborate.”
**Supportive Supervision**	*SW, Org 4*: “I think we can think about, or discuss openly, various reasons they might have not had access to the same opportunities I’ve had for higher education, for mentorship. I think there’s racial dynamics, there’s equity dynamics, maybe lived experience and stigma around that, that has kept people from some of these experiences.”
**Power dynamics**	*CHW, Org 3*: Some time when we say the word social worker…they put guards up thinking negative. So you have to work with them to let them feel comfortable again, trusting…and introducing the worker to them and letting them know…she cares about you…and the purpose they are here…sooner or later the guards [they] come down and allow them to enter in, and then when they get in, they love them, and then they don’t want to let them out of the house. CHW, Org 2: “There’s a lot of opportunity to expand with getting more CHWs in to do the role that I myself am doing without having to pay…the same amount as the social workers, because they are licensed they are…very trained. But we can do that, and still serve and meet people and have there be more equity.”
***Organizational Level Factors***	
**Commitment to Equity**	*SW, Org 2*: “What we’re going to be diving into more as an agency is the social determinants of health. Our community health workers have a pretty solid understanding of the social determinants, and it’s an opportunity…to have our social work staff lean into that a little bit more…because our service delivery model is essentially based on social determinants of health. And so that is a great opportunity that I feel will…enhance the level of collaboration by having both parties have an equal understanding of social determinants of health, and how they impact what we do.”
**Leadership Buy-in**	*CHW, Org 2*: “We don’t have quotas. They allow us the freedom to put in as much effort to make sure that somebody is taken care of as needed. You can spend eight hours with one person and they won’t blink an eye. They’re wonderful.”
**Clear Workflow**	*CHW, Org 3*: “Being a part of [Org 3], you make contact with several of our teams. If you do childbirth, if you do lactation, breastfeeding, and if you do reproductive health…you’re going to come in contact with many members of our team. So that’s why we’re always trying to make sure that trust is built, and we’re building up the entire team.” *CHW, Org 1*: “When we got integrated where we were in a group, where we had a registered nurse, we had a care manager, we had a social worker and a CHW; so we had to figure out what all our roles were going to be and what piece it was going to go to…Right now we’re still working with it…and getting that workflow…where the handoffs [are] going to be, and who’s going to keep a case and who’s going to follow up.”
**Standardized Organizational Training**	*SW, Org 2*: “A lot of the way they’re the same is kind of the way we do intakes with clients. We all follow the same intake script. And so they’re trained in the same way we are, in terms of reaching out for the clients, completing intakes, getting them enrolled on services and then they make referrals. So when it comes to the actual, sit down at the desk, talk to a client over the phone, sit face-to-face with a client at [Org 2], our roles are almost indistinguishable.”
**Shared Documentation**	*SW, Org 4*: “It’s sort of like a joint EMR system that different healthcare systems sign onto and it’s confidential within the systems. And a lot of the client information is shared for care coordination purposes.” *SW, Org 1*: “From an organizational aspect, I would love to see systems more streamlined, so that each one of our people aren’t having to open up four or five different things just to get through the day…especially when it comes to the double documenting.”
**Shared physical space**	*SW, Org 4*: I think over the course of the day, I interact a lot with the housing first team and with SW 22 because we’re all in the same office. Our office is very open plan.
***Societal Level Factors***	
**Sustainable funding**	*SW, Org 1*: I think all of that stuff that you mentioned is very relevant especially nowadays, when a lot of social injustices are coming into the view of the public and even things like health care and with covid, it’s definitely impacted you know our practice…all of that really impacts our work as social workers and CHWs because ultimately that impacts funding, and you know the resources that are available to our client population. *CHW, Org 2*: We’re hoping…to get more community health workers in. Obviously, social workers, their licensed…they’re a little more expensive right, then the community health workers…And we’re understaffed…everybody has needs but if you have an active client, as opposed to you know the five clients that you need to call and the other five clients that you already, they’re calling you and telling you they need these things. You have to address them because they’re already in the system and that pushes the people who are waiting in line further back.

## Data Availability

The data that support the findings of this study are not publicly available. However, deidentified data are available from the authors upon reasonable request to the corresponding author and with the permission of the Coalition for CHW and SW Collaboration.
